# A Local and a National Reproach

**Published:** 1899-12

**Authors:** 


### A Local and a National Reproach.

The fact that one sinner can destroy much good was well illustrated a few years ago by the discredit of Philadelphia medical diplomas on account of the Buchanan diploma-mill. A similar experience seems now likely to fall to Chicago through the disreputable "Independent Medical College," and its predecessors and successors. It appears, according to the *Times* of India, quoted by a correspondent in the *Indian Medical Record*, that one "Professor" Van Noppen, a coadjutor of the notorious Armstrong and others in this fraudulent concern, has traveled far and wide in India, "leaving a trail of diplomas behind him," and Bombay, Calcutta, Allahabad and other Indian cities can each rejoice in the possession of a coterie of "Chicago graduates." The *Times* of India calls the Indian Government's attention to some official appointees in the plague districts, especially to one or more who append the "M.D." (Chicago) to their names, and suggests an inquiry. It says: "In view of the discredit which has been cast upon Chicago degrees in general, it would be reassuring to the public if it were officially stated that a diploma which has received implied approval in the pages of a Government publication was bestowed by a duly qualified examining body." The law enacted by the last Legislature affecting these diploma-mills was passed none too soon. Their existence was not only a reproach to Chicago and the nation at large, but has been an actual damage to its respectable medical institutions.—*The Journal.*

THE Russian Government has just forbidden the further sale of a supposed herbal remedy that, owing to extensive advertising, was in wide use among the people. Under the name of Ephedra Vulgaris the remedy had hitherto escaped the rigid law against secret remedies in Russia, its title being its true botanical name. A large amount of the stuff ready for shipment was confiscated and proved to be ordinary hay. Pound packages were being sold in large numbers at from three to five roubles ($1.50 to $2.50) a pound.—*Medical Sentinel.*

